# Histopathologic risk factors in oral and oropharyngeal squamous cell carcinoma variants: An update with special reference to HPV-related carcinomas

**DOI:** 10.4317/medoral.20184

**Published:** 2014-06-01

**Authors:** Samir K. El-Mofty

**Affiliations:** 1 DMD, MS, PhD. Professor. Washington University School of Medicine, Department of Pathology and Immunology

## Abstract

Accurate identification of the microscopic risk factors of oral and oropharyngeal (OP) squamous cell carcinomas (SCC) and their morphologic variants is of at most importance, as these generally determine treatment modalities, prognosis and overall patient outcome. The great majority of oral and oropharyngeal squamous cell carcinomas are microscopically described as kerartinizing squamous cell carcinoma (KSCC). They bear certain resemblance to keratinizing stratified squamous epithelium. Tobacco habits and excessive consumption of alcoholic beverages have been considered to be the main etiologic agents in these carcinomas. The tumors occurred in older patients more commonly affected the oral tongue and floor of the mouth with well established morphologic risk factors including tumor grade, pattern of invasion and perineural involvement. Within the last 30 years however, the advent and expanding prevalence of high risk human papillomavirus (HPV) as an important etiologic agent for head and neck squamous cell carcinoma, particularly in the OP, has resulted in a significant change in the established morphologic criteria for risk assessment. The majority of HPV relate carcinomas of the OP are nonkeratinizing squamous cell carcinoma (NKSCC). These tumors are found to be more responsive to treatment with a favorable patient outcome and good prognosis. Consequently, alterations in treatment protocols aimed at de-escalation are currently being evaluated. More recently, other morphologic variants that are HPV positive are reported with increasing frequency in the OP and other head and neck sites. As a result, several clinical and pathologic questions have emerged. Importantly, whether the virus is biologically active in these tumors and involved in their pathogenesis, and second, what are the clinical implications with regard to patient management and outcome in the HPV-related variants. Examples of HPV-related squamous cell carcinoma variants that will be addressed here are: basaloid squamous cell carcinoma (BSCC), undifferentiated carcinoma (UCa), papillary squamous carcinoma (PSCC) and small cell carcinoma. Some studies have suggested favorable prognosis in some variants, analogous to that of the (NKSCC), while others showed poorer outcome. So far the number of studies on this subject is limited and the number of cases evaluated in each investigation is few. Because of that, it is prudent at this stage, not to alter management protocols as a result of identification of HPV in these variants and to await additional information

** Key words:**Histopathologic risk-factors, oral cavity, oropharynx, squamous cell carcinoma variants, keratinizing squamous cell carcinoma, nonkeratinizing squamous cell carcinoma, HPV, basaloid squamous cell carcinoma, undifferentiated carcinoma, papillary squamous cell carcinoma, small cell carcinoma.

## Keratinizing squamous cell carcinoma (KSCC); morphologic risk factors

Traditionally KSCCs have been graded according to their state of differentiation and resemblance to normal squamous epithelium into well, moderate and poorly differentiated variants. The grading system which was originally proposed by Broder (1) was based on the amount of keratin production and pleomorphism of the tumor cells. However, because the morphologic features can vary considerably from area to area within the same tumor, the Broder grading system was found to lack significant prognostic value. Several authors suggested that more useful prognostic information may be deduced from the invasive fronts of the tumors where the deepest and presumably more aggressive cells reside (2,3). According to this system five histologic features are graded and assigned scores from one to four. The scores for all the variant are summed to provide a total malignancy score for a particular tumor. The parameters used are: degree of keratinization, nuclear pleomorphism, number of mitosis per high power field (HPF), pattern of invasion and inflammatory lymphoplasmacytic host response. The highest scores are given to tumors with low or no keratinization, extreme nuclear pleomorphism, >5 mitosis/HPF, marked cellular dissociation in small nests and single cells and no host response.

A newer risk model was developed by Brandwein-Gensler and associates (4-6) as an extension and modification of prior multivariable histologic systems analyzing the advancing tumor front. In this model a risk category is assigned by examining the resection specimen of the primary tumor and quantifying 3 significant histologic variables; 1- Pattern of invasion at the advancing tumor edge (PI). The different types of patterns are described as; pushing border, finger like growth, large islands, small islands or distant satellites. 2- Perineural invasion (PNI) involving either small nerves or large ones (> 1mm). 3- Lymphocytic host response at the advancing tumor edge described as strong, intermediate or weak. The risk model classified the tumors, according to a point score into low, intermediate, and high risk groups which correlated significantly with local recurrence rates and overall survival in a cohort of patients with head and neck squamous cell carcinoma (5). The prognostic value of this risk model was further validated in patients with low-stage (Stag I/II) oral squamous cell carcinoma. The model was significantly predictive of loco-regional recurrence and disease specific survival (6). Based on these observations it was suggested that some low stage oral SCC that would traditionally be treated surgically may benefit from adjuvant radiotherapy if classified in the high risk group. Tumor thickness in oral KSCC has also been used as a parameter for outcome prediction in T1 carcinomas. However, it was found that the cut-off tumor thickness varied by anatomic site. In addition, it predicted lymph node metastasis and survival but not local recurrence.

## HPV-Related squamous cell carcinoma 

During the last 30 years accumulating epidemiologic and clinical evidence has shown that high risk human papillomavirus (HPV) is a major etiologic factor in a subset of head and neck squamous cell carcinomas. These tumors have distinct clinical, microscopic and molecular features. The greatest majority of the carcinomas occur in the OP, particularly the palatine and lingual tonsils areas. They are characterized by younger age at onset (7), weak or no association with alcohol and tobacco use but strong association with sexual behavior, particularly oral sex (8,9). Despite a characteristic early lymph node metastasis, HPV-related oropharyngeal squamous cell carcinomas are associated with significantly better treatment outcome and patient survival (10-12).

**Figure 1:**
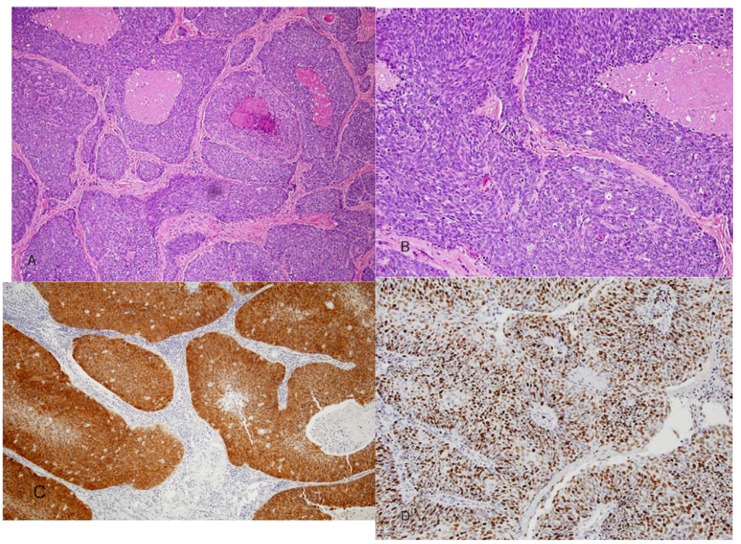
A) Nonkeratinizing squamous cell carcinoma. Sheets and large nests of tumor cells are seen with well defined borders and central comedo necrosis. B) Higher magnification showing small ovoid and spindle shaped tumor cells with hyperchromatic nuclei and indistinct cell bordrs. C) Strong and diffuse p16 immunostaining, both nuclear and cytoplasmic. D) Ki-67stain showing high labeling score of more than 80%.

**Figure 2:**
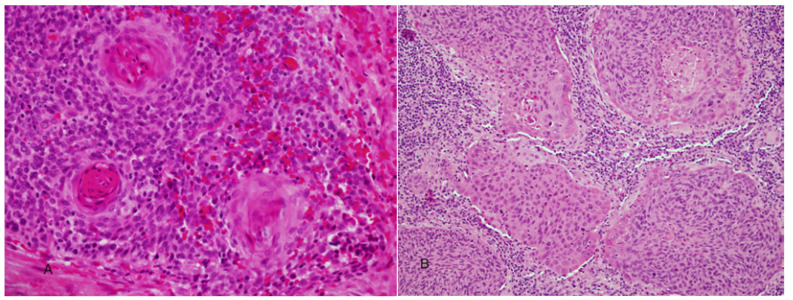
A) Nonkeratinizing squamous cell carcinoma with focal areas of keratinocytic maturation (hybrid variant). B) Nonkeratinizing hybrid variant with peripheral partial maturation of basaloid cells.

Microscopically, HPV-related squamous cell carcinoma of the OP are distinguished by a nonkeratinizing morphology (7,11,13). The tumors are characterized by relatively monomorphic, ovoid and spindle-shaped basaloid cells with indistinct cell border (Fig. 1). The nuclei are hyperchromatic with high nuclear-to-cytoplasmic ratio. The cells form sheets, nests and cords with sharply defined borders. Excessive mitosis and apoptosis as well as comedo-type necrosis are observed. These carcinomas also show a distinct immunohistochemical profile namely, a strong and diffuse p16 reactivity, very high Ki-67 labeling scores (Fig. 1) and negative or week staining with p53. A hybrid form of NKSCC in which the basaloid tumor cells show focal and partial keratinocytic maturation has been described by El-Mofty *et al*, (13) (Fig. 2). This hybrid variant was shown to be HPV related and to also have a favorable clinical outcome (11).

p16 over expression is now considered a surrogate marker for HPV-related NKSCC of the OP (7,14). p16 is a cell cycle protein associated with tumor suppression by the retinoblastoma pathway. It inhibits hyperphosphorilation of retinoblastoma protein (pRb) thus preventing its dissociation from the transcription factor E2F and the subsequent progression of the S phase of the cell cycle (15,16). HPV E7 oncoprotein interacts with pRb active form resulting in its functional inactivation. The paradoxical overexpression of an inhibitory protein in actively replicating neoplastic cells is thought to result from feed-back control secondary to pRb deregulation (17,18).

It has been shown that accurately identifying the nonkeratinizing morphologic features and its hybrid variant in oropharyngeal squamous cell carcinomas can be used as predictor of favorable clinical outcome (19). The nonkeratinizing morphology is significantly more likely to be HPV and p16 positive than KSCC, and to have better overall survival (OS) and disease specific survival (DSS) with a p value of<0.001 and 0.01 respectively (19). It was also shown that p16 overexpression in oropharyngeal SCC is associated with HPV positivity and has significantly better patient outcome (20). The pattern of p16 immunohistochemical staining that correlates with transcriptionally active HPV and better patient outcome is strong and diffuse both nuclear and cytoplasmic in more than 75% of the tumor cells (Fig. 1) (20,21).

Because of the observed disparity in clinical outcome of squamous cell carcinomas consequent to their HPV status, there are ongoing clinical investigations to test the efficacy of altering treatment paradigms in documented HPV- related tumors. Multi-institutional clinical trials are underway to determine the feasibility of de-escalated treatment modalities in these cases. It is therefore of importance that HPV status should be reliably and accurately determined. Detection p16 overexpression by immunohistochemistry is the most commonly used technique for this purpose. However in rare occasions p16 is not specific. Another common detection method is in situ hybridization (ISH) for HPV DNA. While highly specific it is not very sensitive. On the other hand, PCR analysis for HPV DNA is highly sensitive but not necessarily specific. Detecting viral DNA by PCR does not indicate whether the virus is trancriptionally active (driver) or a bystander (passenger).

E6/E7 mRNA expression is considered the “gold standard” for identification of clinically significant HPV infection in tumor specimens. Reverse transcriptase polymerase chain reaction (RT-PCR) and real-time quantitative RT-PCR (RT-qPCR) are the main methods used for detection and quantitation of E6/E7 mRNA (22,23). More recently, an in situ hybridization method has been developed for detection of transcriptionally-active HPV in head and neck squamous cell carcinomas. ISH for E6/E7 mRNA is a slide-based chromogenic assay that has been developed under the name RNA scope (Advanced cell diagnostics, Inc., Hayward, CA) (24,25). Results from HPV E6/E7 mRNA ISH were found to be highly concordant with p16 immunohistochemistry and RT-qPCR (24,25).

A small minority of HPV+ OP SCC show atypical clinical behavior following treatment including poor OS, DSS and Disease free survival (DFS) (12,26,27). Some cases may be associated with multicentric disease in the mucosa of the upper aerodigestive tract excluding the oral cavity. The reported sites of involvement include the larynx, nasopharynx and sinonasal tract (28). Also, on rare occasion HPV+ OP SCC was reported to have multiple distant metastasis to unusual sites including the skin brain, gastrointestinal tract and intra-abdominal lymph nodes (28,29) The interval from completion of therapy to onset of distant metastasis ranged from 4- 11 (28) and 2-52 (29) months with a median of onset 7 and 18 months respectively (28,29).

The exact causal mechanisms involved in poor clinical outcome and patient survival in a minority of HPV positive SCC of the oropharynx are not clear. However, certain relevant molecular profiles have been proposed. These include over-expression of Bcl2, EGFR, and BclxL, (27,30,31). EGFR is a transmembrane tyrosine kinase, its function affects cell cycle progression, apoptosis, angiogenesis, tumor cell mobility and metastasis. Over expression of EGFR was shown to be associated with poor OS and DFS in HPV +, p16 + SCC of the oropharynx (30,31). Similarly over-expression of the antiapoptotic proteins Bcl2 and BclxL was shown to be associated with poor OS and DFS as well as DSS in HPV + OPSCC (27,31) these proteins are known to confer resistance to chemotherapy and radiation therapy of head and neck SCC. It has also been documented that patients who continue to smoke after treatment have a significantly worse DSS than those who were past or never smokers (12,31).

## HPV Related squamous cell carcinoma of the oral cavity

Unlike the OP the oral cavity proper is a rare site for HPV related SCC. El-Mofty and LU (7), using PCR and p16 immunostain, have shown in a cohort of young patients who are 40 years of age or younger that the prevalence of HPV related SCC of the palatine tonsils was (91%) 10/11 while in the oral cavity the prevalence was (0%) 0/15. However, considerable variations in the prevalence of HPV in oral SCC have been reported in the literature. These variations may be attributable to differences in specificity and sensitivity of the tests used and whether the identified virus is a “driver” or a “passenger” in these tumors. Prevalence may also vary by geographic distribution of the studied cases. A range of between 0%-11% has been reported (7,32-35). HPV+ oral SCC, unlike those in the OP, do not exhibit nonkeratinizing morphology. The significance of HPV in oral SCC and its relationship to treatment outcome and patient’s survival is currently not known.

## HPV-Related SCC variants

The great majority of HPV relate SCC of the OP is nonkeratinizing as described above. However, more recently increasing numbers of variants of squamous cell carcinoma, that are HPV-positive, are reported in the oropharynx as well as in other head and neck sites. As a result, several clinical and pathologic questions have emerged. Importantly, whether the virus is biologically active and involved in the pathogenesis of these tumors, and whether there are clinical implications with regard to patient outcome and treatment modality changes that may be needed in HPV related variants. Examples of HPV-related squamous cell carcinoma variants that will be addressed here include: basaloid squamous cell carcinoma, undifferentiated carcinoma, papillary squamous carcinoma, small cell carcinoma and keratinizing squamous cell carcinoma. Some investigations have suggested favorable prognosis in some HPV positive variants, analogous to that of the nonkeratinizing carcinoma, while others showed poorer outcome. So far the number of studies on this subject is limited and the number of cases evaluated in each investigation is few. Because of this, it is prudent that at this stage, not to alter management protocols as a result of identification of HPV in these variants and to await additional studies.

## Basaloid squamous carcinoma (BSCC)

**Figure 3:**
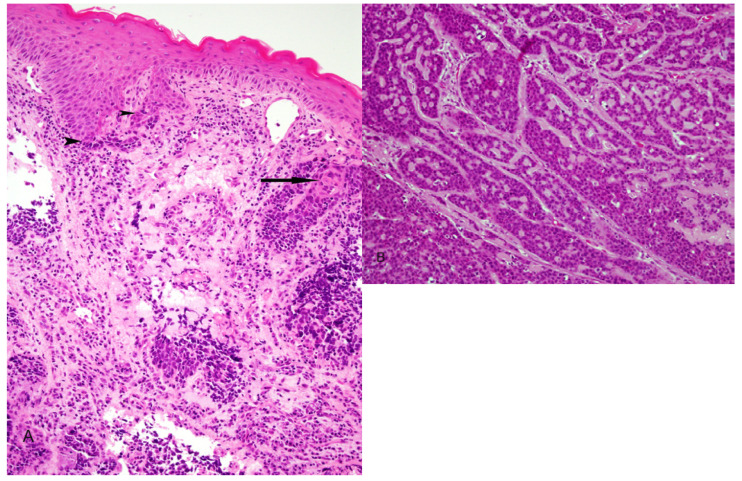
Basaloid squamous cell carcinoma. A) Illustrates biphasic pattern with conventional dysplastic squamous surface component associated with basaloid elements (arrow heads) and conventional squamous cell carcinoma intimately associated with basaloid component (arrow). B) Closely packed basaloid cells forming a "jigsaw puzzle" appearance. Microcystic cribriform-like pattern is also observed.

In the upper aerodigestive tract, BSCC is rare variant of conventional SCC. It occurs more commonly in the hypopharynx and larynx and less frequently in the oropharynx. Like SCC the tumor is typically associated with traditional risk factors like tobacco smoking and alcohol abuse. It is generally considered a high grade malignant neoplasm with poor prognosis. Microscopically, it is characterized by a biphasic pattern. A basaloid component intimately associated with elements of keratinizing SCC. The basaloid cells are small, crowded with hyperchromatic round nuclei and scant cytoplasm. They form sheets and lobules that produce a “jigsaw puzzle” growth pattern with cystic spaces containing PAS –positive myxoid material. Stromal hyalization may be present (Fig. 3). The squamous component is either surface SCC or severe dysplasia in addition to focal abrupt squamous differentiation within the basal cell areas (Fig. 3). These microscopic features are distinct from but may be confused with those of NK SCC as described above.

A causal relationship between HPV and some cases BSCC has been documented in the upper aerodigestive tract. Chernock *et al*, (36) using ISH for HPV DNA and p16 immunohistochemistry found that 75% of orophryngeal BSCC are HPV related while none (0%) of 16 laryngeal/hypopharyngeal tumors were positive (36). Using PCR for HPV 16 DNA as well as p16 immunostaining, Begum and Westra (37) documented HPV in 76% of oropharyngeal BSCC and in 6% of tumors in non-oropharyngeal sites. In both studies (36,37) the overall survival was significantly better in HPV positive tumors than in the HPV negative ones with *p*< 0.05 and <0.0001 respectively.

## Undifferentiated carcinoma (lymphoepithelial, nasopharyngeal type)

In the head and neck undifferentiated carcinoma (UDCa) is best recognized in the nasopharynx where it is referred to by a variety of names including; lymphoepithelioma, nasopharyngeal type undifferentiated carcinoma and nonkeratinizing undifferentiated carcinoma (WHO type III). Microscopically identical carcinomas are also identified in the oropharynx. While undifferentiated carcinoma of the nasopharynx has a strong etiologic relationship to Epstein-Barr virus (EBV) (38), undifferentiated carcinoma of the oropharynx has recently been shown to be predominantly HPV and not EBV-related (39,40).

**Figure 4:**
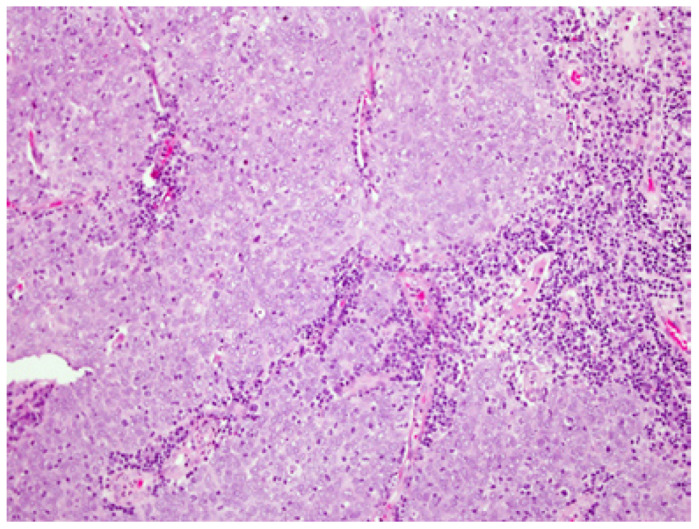
Undifferentiated (lymphoepithelial) carcinoma. Undifferentiated epithelial cells forming a syncytium and intermingled with lymphocytes and plasma cells. The tumor cells have large vesicular nuclei.

The microscopic features of oropharyngeal undifferentiated carcinoma are indistinguishable from those of the nasopharyngeal type as defined by the WHO. The tumors are composed of solid sheets, trabeculae, nests and single neoplastic epithelial cells intimately intermingled with lymphocytes and plasma cells. The epithelial tumor cells are large with indistinct cell borders forming a syncytium (Fig. 4). The nuclei are round to oval and vesicular with large central nucleoli. HPV DNA was identified in these tumors by PCR and ISH. Evidence for biologic activity of the virus was demonstrated by p16 over- expression (39,40). The 3 year OS was found to be 55%, while the DSS was 100% (39). No tumor recurrence was observed during a median follow up period of 23 months (40). The patient’s outcome in HPV positive undifferentiated carcinoma of the OP is generally favorable and comparable to that of the nonkeratinizing HPV-related squamous cell carcinoma.

## Papillary squamous cell carcinoma 

Papillary squamous cell carcinoma (PSCC) is a poorly understood variant of SCC of the upper aerodigestive tract that is often confused with other exophytic mucosal malignancies such as verrucous carcinoma and squamous cell carcinoma with verrucous features. As defined by the WHO, PSCC is characterized by a predominant papillary growth pattern with thin fibrovascular cores covered by immature basaloid cells or dysplastic cells with minimal or no keratinization. PSSC is generally believed to have a better prognosis than conventional squamous cell carcinoma.

Carcinomas with papillary growth pattern occurring in the uterine cervix, vulva and penis are commonly associated with High risk HPV particularly type 16. Very limited number of studies has so far investigated the prevalence and significance of HPV in PSCC of the head and neck (41,42). Jo *et al*. (41) found that 15 of 31 cases of PSCC of the head and neck were both p16 and HPV ISH positive. The majority of those (11 cases) were oropharyngeal. Patient outcome in HPV positive and negative tumors was not adequately investigated.

**Figure 5:**
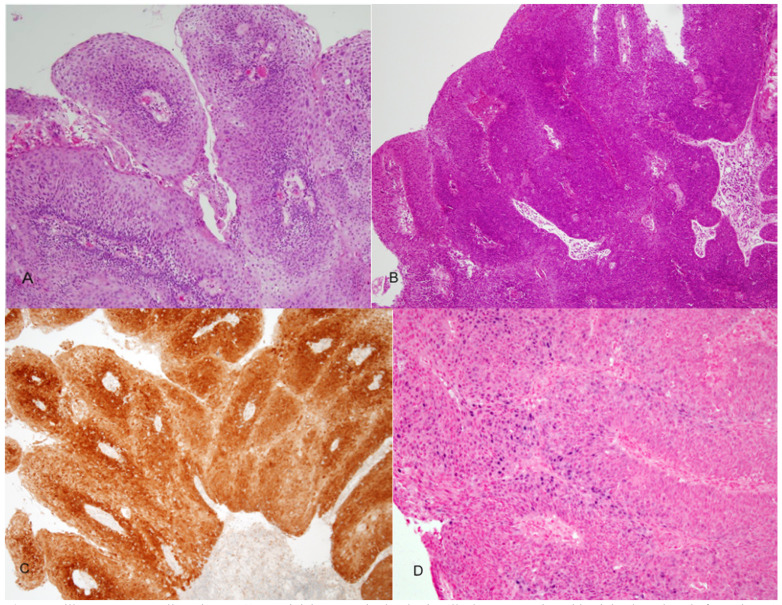
Papillary squamous cell carcinoma. A) Keratinizing type, the dysplastic cells show maturation with minimal parakeratin formation. B) Nonkeratinizing type with immature basaloid cells. C) strong and diffuse p16 staining. D) Positive ISH for high risk-HPV DNA (blue nuclear staining).

In a recent study (42) we reviewed 48 cases of PSCC of the head and neck, 7 in the oral cavity 19 oropharynx and 22 in the larynx. Two morphologic types were identified: (1) a keratinizing type (K) in which the dysplastic epithelium showed maturation trend with minimal surface parakeratin, and a nonkeratinizing (NK) type in which the papillae were covered with immature basaloid cells (Fig. 5). An HPV relationship was identified by p16 immunoreactivity, HPV ISH and E6 and E7 mRNA ISH in a number of tumors (Fig. 5). The majorities of these were found in younger patients, occurred more commonly in the OP, had NK morphology and were less likely to be p53 positive. Disease specific survival (DSS) was favorable in all cases. The 5 years DSS for p16 positive and p16 negative cases was 80% and 70 % respectively. No statistically significant difference in OS, DSS or disease-free survival (DFS) was found regarding tumor site, morphologic type or HPV relationship. However, a trend towards better DFS was seen in patients with p16 positive/HPV positive tumors (42).

## Small cell (neuroendocrine) carcinoma

An association between HPV and neuroendocrine carcinoma of the oropharynx was shown in two recent studies (43,44). Bishop and Westra (43) found that 5 of 9 cases of oropharyngeal small cell carcinoma (poorly differentiated neuroendocrine carcinoma) were HPV-related. In 4 of these cases the tumors were associated with typical HPV-related SCC. All 5 tumors were p16 positive by immunohostochemistry and HPV positive by ISH. All cases showed a characteristic neuroendocrine immunophenotype, including reactivity to synaptophysin and /or chromogranin and CAM 5.2 and were negative for CK 5/6. Three of the 5 patients died within 15 month of diagnosis (mean 10 months) with widely disseminated disease. Such outcome is in sharp contrast to the typical HPV-related nonkeratinizing SCC which is associated with a 3-year survival rate of up to 80% (12). Kraft *et al*. (44) reported 8 oropharyngeal neuroendocrine carcinomas that were HPV-related. Disease recurrence occurred in 5 of 6 patients with available clinical follow-up, with 3 developing metastasis to bone, lung, pleura, adrenal gland and pancreas.

HPV-related small cell carcinoma of the oropharynx shares common features with small cell neuroendocrine carcinoma of the uterine cervix. Both are associated with high risk HPV, commonly coexist with non-small cell squamous cell carcinoma and share the same aggressive clinical behavior with early distant metastasis and poor overall survival (43-46).

## HPV-Related keratinizing squamous cell carcinoma of the oropharynx

Classical KSCC of the OP is becoming much less frequently encountered in some parts of the world including the United States and some European countries. A very small minority of these are HPV related. In a recent study we have found that only 7 of 54 (13%) KSCC of the OP overexpressed p16. HPV E6/E7 RNA ISH was positive in 5 tested cases. The OS and DSS was significantly better in the p16 positive than the p16 negative KSCC cases (*p*=0.01 and 0.04) respectively (47).

## Discussion

The histopathologic features of keratinizing SCC of the upper aerodigestive tract have been used by Pathologists as tools for prediction of tumor behavior and patient prognosis. Consequently, Clinicians have relied on this information for determining treatment modalities to be used in each particular case. The greatest majority of these keratinizing neoplasms shared morphologic risk features that are used to classify them in a range of grades, from low to high. Less frequently, variant forms of KSCC such as BSCC, PSCC, adenosquamous carcinoma, spindle cell carcinoma and UCa are encountered. These variants have distinct morphology and clinical behavior and are not graded.

Within the last few decades an HPV related nonkeratinizing variant of SCC was identified. The majority of which occurred in the oropharynx. The neoplastic cells in these tumors show an immature basaloid appearance and are mitotically active. Such phenotype is not limited to oropharyngeal sites but it is also observed in other HPV related tumors in variety of sites including the sinonasal tract, larynx the and anogenital sites (48,49).The exact molecular mechanisms underlying the expression of this specific histopathologic phenotype are not clear. It is possible that interactions between HPV oncoproteins and cell cycle mediators may play a role. It is known that high risk HPV oncoproteins, particularly E6 and E7, interfere with Rb and p53 functions leading to cell cycle progression, cell immortalization, suppression of apoptosis and essentially uncoupling of proliferation and maturation. It is conceivable that these changes may be responsible for the characteristic immature basaloid appearance of the tumor cells. Chromosomal instability is another property of HPV oncogenesis. It is likely that additional mutations may be responsible for the development of those rare microscopic SCC variants discussed above.

As the numbers of reported HPV positive head and neck squamous cell carcinoma variants are increasing, particularly in the oropharynx, it is of importance to establish the status of the virus in the tumors cells and to distinguish between a causal agent “driver” and a bystander “passenger”. Currently, the number of cases of SCC variants with established HPV causal relationship is still limited. Based on these studies, there is some evidence to suggest that variants such as BSCC, undifferentiated carcinoma and PSCC may have favorable prognosis similar to that of conventional NKSCC and better than their HPV negative counterparts. On the other hand, HPV related small cell carcinoma has been shown to be associated with early distant metastasis and poor overall survival. More over a minority of NKSCC of the oropharynx have been recently found to be associated with atypical clinical behavior following treatment, including poor OS, DSS and DFS (12,26,27).It is thus too early to reach a definite conclusion regarding the biologic behavior of HPV-related SCC morphologic variants. Surgeons and oncologists should be warned that HPV status in these tumors should not be used, at this time, as a justification for considering de-escalation of conventional therapy.
